# Epidemiological, Clinical and Laboratory Features of Strongyloidiasis in 69 Attendees at a French Outpatient Clinic

**DOI:** 10.3390/pathogens12080983

**Published:** 2023-07-27

**Authors:** Jean-François Magnaval, Judith Fillaux, Richard Fabre, Sophie Cassaing, Alexis Valentin, Xavier Iriart, Antoine Berry

**Affiliations:** 1 Service de Parasitologie Médicale, Faculté de Médecine, Université de Toulouse, 31000 Toulouse, France; 2 Service de Parasitologie-Mycologie, Hôpital Purpan, Centre Hospitalier Universitaire de Toulouse, TSA 4003, 31059 Toulouse, France; 3 DENDRIS, 335 Rue du Chêne Vert, 31670 Labège, France; 4 FLAMES/RESTORE (Inserm UMR 1301/CNRS UMR 5070/EFS), Université de Toulouse, 31100 Toulouse, France; 5 PHARMA-DEV (UMR 152), Institut de Recherche Pour le Développement, Université de Toulouse, 31062 Toulouse, France; 6 Institut Toulousain des Maladies Infectieuses et Inflammatoires (UMR “Infinity”, Inserm/CNRS/Université de Toulouse III), 31024 Toulouse, France

**Keywords:** strongyloidiasis, southwestern France, epidemiology, clinical picture, eosinophilia, *Strongyloides ratti*, ELISA

## Abstract

The present retrospective study analyzed the characteristics of strongyloidiasis in patients who were diagnosed at the Outpatient Clinic of the Department of Parasitology-Mycology, Toulouse, France. Sixty-nine file records were included in the study on the basis of a positive stool examination that used Baermann’s method. The prominent epidemiological findings were the presence of former immigrants from Italy or Portugal, veterans from the 1st Indochina war, and autochthonous cases. Almost 1/4 of the patients were asymptomatic. Manifestations of skin allergy were the main clinical feature. Blood eosinophilia was present in 76.8% of the patients, and serum total IgE was ≥150 kIU/L in 79.7%. Immunodiagnosis was achieved from 1990 to 2001 by indirect immunofluorescence (IFAT) that was then replaced with ELISA, both methods using *Strongyloides ratti* filariform larvae. ELISA was found to be similar to IFAT in terms of specificity but exhibited a greater sensitivity. Patients were primarily treated with albendazole or ivermectin beginning in 1993. Forty-eight patients attended the follow-up consultation. Kinetics of the clinical picture and blood eosinophilia were found to be the most convenient parameters to assess the efficacy of anthelmintic therapy. In conclusion, strongyloidiasis remains a neglected disease in Southwestern France. The resolution of clinical features along with the kinetics of eosinophilia appeared to be the most appropriate parameters to check during the posttreatment follow-up.

## 1. Introduction

Strongyloidiasis is a helminth infection mostly due to the nematode *Strongyloides stercoralis*, the parthenogenetic females of which are embedded in the duodenal and ileal mucosa of the host’s intestine. The lifecycle of *S. stercoralis* is complex. Usually, the infection is acquired when infective (filariform) larvae penetrate the skin following contact with soil contaminated with feces. Next, they go through the lungs, follow the airways and then the esophagus, and finally reach the small bowel. In the mucosa, parasitic females produce by parthenogenesis eggs that transform into rhabditiform larvae in the intestine. These larvae are passed in stools, molt in the soil, and transform into infective, filariform larvae. In the soil, some larvae can undergo so-called indirect development. This process results in the appearance of free-living male and female adult worms. After mating, the female worms release eggs which hatch into rhabditiform larvae which become infective filariform larvae. Moreover, an autoinfection route exists that makes *S. stercoralis* unique among the helminths infecting humans. In chronic infection, a variable number of rhabditiform larvae develop into filariform larvae within the intestinal lumen. These infective forms pass through the intestinal wall or the skin of the anal area and then follow in the human organism the above-depicted pathway. Due to this autoinfection route, strongyloidiasis usually is a permanent infection that can persist for decades [1]. Recent genetic studies on *S. stercoralis* strains isolated from dogs have demonstrated that these companion animals carry two populations, possibly different species of *Strongyloides*. One population appears to be dog specific, but the other one is shared with humans [2]. These findings suggest possible zoonotic transmission of *S. stercoralis* to humans.

Clinical manifestations differ according to the stage of infection. The acute form corresponding to a recently acquired infection is usually observed in travelers returning from endemic areas. In the chronic phase, many patients are asymptomatic. This status can last for decades, and clinical manifestations can occur long after the initial infection. The classic picture comprises abdominal pain, intermittent diarrhea, and various signs or symptoms of skin allergy (chronic-to-permanent pruritus, pruriginous rashes, or urticaria) [3]. In some patients, an urticarial rash may migrate and develop into a serpiginous or linear, erythematous, recurrent subcutaneous trail. This pathognomonic sign corresponds to the subcutaneous passage of a filariform larva during the autoinfection cycle and has been termed *larva currens* [4]. Hyperinfection syndrome (HS) originates in the dysregulation of the autoinfection process, leading to many filariform larvae that penetrate the gut wall, then travel to the lung and finally reenter the intestine. Exacerbation of this process results in disseminated strongyloidiasis (DS), which is characterized by larval migration away from the lung and gastrointestinal tract into other organs or tissues [5]. Since migrating larvae from the gut carry bacteria of the intestinal flora, secondary bacteriemia and bacterial meningitis can follow. HS and DS are life-threatening conditions in which the fatality rate is near 90% [6]. Predisposing risks for HS/DS are well identified and include debilitating conditions (alcoholism, disseminated carcinomas, malnutrition, or tuberculosis), infection with HTLV-1 virus, and treatments with anticancer or immunosuppressive drugs that are used in oncology or transplantation practice. Corticosteroids are recognized as having a strong association with the occurrence of HS/DS, regardless of the dose or the route of administration. It has been hypothesized that corticosteroids stimulate the production of ecdysteroid-like substances that act as molting signals for rhabditiform larvae [7]. Due to the rise in migrations from areas endemic for strongyloidiasis to developed areas or to the presence of autochthonous strongyloidiasis in certain industrialized countries, the incidence rate of HS/DS is increasing among patients who have access to high-grade medicine, including anticancer or immunosuppressive chemotherapy or corticosteroids, and therefore, HS/DS represents a worldwide growing threat [6]. The intensive use of corticosteroids during the COVID-19 pandemic has represented a further risk factor for HS/DS in patients from areas endemic for strongyloidiasis [8].

In the chronic phase, the presence of blood eosinophilia and increased levels of serum total IgE are common [1], but both abnormalities may be absent in up to 25% of patients [9]. When the blood eosinophil count was tested during weeks or months in patients presenting with longstanding eosinophilia, great variations in the results were observed, so the resulting curve of eosinophilia displayed an undulating appearance [10,11].

Laboratory diagnosis of strongyloidiasis relies upon an array of methods, including microscopy examination of stools, agar-plate culture of feces samples, serodiagnosis and, more recently, molecular methods. Due to the intermittent shedding of rhabditiform larvae in stools, conventional microscopy (direct examination plus concentration methods) on a single specimen exhibited poor sensitivity, ranging from 15% [12] to 30% [13]. A single examination by the specific Baermann’s method detected 33% of the cases [12,14], and eight consecutive tests were required to increase the sensitivity rate up to 66% [14]. Agar-plate culture of stools detected *S. stercoralis* larvae in 89% to 94% of patients [15]. Immunodiagnostic techniques have addressed the issue of the poor sensitivity of microscopy examination of stools at the price of a moderate lack of specificity. By the end of the 1960s, an indirect immunofluorescence antibody test (IFAT) using *S. stercoralis* larvae as the antigen was suggested for use as a routine method [16]. Subsequently, filariform larvae of *S. ratti* were tested. IFAT using this heterologous antigen or homologous *S. stercoralis* larvae displayed similar performances [17]. Depending on the dilution used for the cutoff value, the sensitivity (Se) ranged from 95% to 98% [15,17,18] and the specificity (Sp) from 97% to 98% [18,19]. ELISA using purified extracts from *S. ratti* or *S. stercoralis* larvae or recombinant antigens (Ss-NIE or Ss-IR [20]) has succeeded IFAT as a routine technique for the immunodiagnosis of *S. stercoralis* infection. The Se displayed by commercial kits based upon these antigenic reagents ranged from 91% (*S. ratti*-based ELISA) to 99% (Ss-NIE/Ss-IR ELISA), and the Sp varied between 94% (idem) to 99% (ibid.) [20,21]. Molecular methods have an intrinsic high specificity, along with recognized reliability and reproducibility. Moreover, these techniques are operator-independent with respect to the interpretation of the results and, therefore, overcome the worldwide growing issue of the availability of well-trained technicians for microscopy diagnosis. Se ranges from 17% to 100%, and Sp ranges from 76.7% to 100%, according to the nature of the genetic marker, the primer used, and the type of PCR [22]. In most studies, DNA was extracted from fecal specimens, but the use of urine and particularly of serum displayed interesting perspectives [23].

To date, strongyloidiasis remains a neglected helminthiasis for which a worrisome knowledge gap subsists concerning the characteristics of chronic infection [24]. Therefore, the aim of the present study was to assess retrospectively the epidemiological, clinical, and laboratory features of strongyloidiasis in patients who were diagnosed between 1991 and 2013 at the Outpatient Clinic of the Department of Parasitology-Mycology, Toulouse University Hospitals, France.

## 2. Patients and Methods

### 2.1. Criteria for Inclusion in the Study

For inclusion in the study, patients had to present with a positive stool examination for *S. stercoralis* larvae, along with a complete set of data (demographic, epidemiological, clinical, and laboratory) that had been collected at their first visit to the clinic. According to these criteria, 69 file records were extracted from the department database. The covered period ranged from 1991 to 2013. Forty-eight patients attended the posttreatment follow-up consultation.

### 2.2. Diagnosis of Strongyloidiasis

At the Outpatient Clinic of the Department of Parasitology-Mycology, the diagnosis of strongyloidiasis relied primarily upon stool examination. The results from specific immunodiagnosis were considered only as additional diagnostic information. Patients were investigated by two authors (JF or JFM).

For any patient who was diagnosed with chronic strongyloidiasis, a detailed questionnaire inquiring about demographics (age and sex), current and past occupation, origin or travel or stays outside France, and place of residence was recorded along with the patient’s medical history. As much as possible, the time interval between the onset of manifestations and the attendance at the clinic was recorded in weeks. The clinical picture was evaluated on general examination and quantified by a rating procedure. That is, any sign or symptom was rated as 2 if present and 0 if absent. At the follow-up consultation, the rating was 3 if an increase was noticed, 2 if the expression of the parameter was stable, 1 if it was reduced, and 0 if it had vanished. All these data were included in the patient’s file record.

### 2.3. Laboratory Methods

Total and differential blood counts were performed routinely on various autoanalyzers in the Department of Hematology, and the assay for serum total IgE was routinely carried out in the Department of Immunology.

#### 2.3.1. Microscopy

Upon appointment request for consultation, patients were instructed to bring with them whole stools, preferably voided on the morning of attendance to the clinic to ensure availability of a fresh sample. According to the legal requirements of the French Ministry of Health, stool examination by microscopy included direct examination plus two concentration methods. In our department, ZnSO_4_ flotation [25] was primarily used and then replaced with the Ovatec *Plus*^™^ kit (Synbiotics, Lyon, France). Diphasic merthiolate-iodo-formol (MIF) concentration was the other technique employed [26]. Baermann’s method [25] was systematically executed for every stool examination.

#### 2.3.2. Immunodiagnosis: Maintenance of a *Strongyloides ratti* Line

From the end of the 1980s, the department used *S. ratti* as a source of antigenic reagents. Employed either in IFAT [17] or in ELISA [27], this heterologous antigen proved convenient. A *S. ratti* line was maintained by serial passages in male Wistar rats. Animals were hosted in the Department Laboratory Animal House in accordance with regulations R.214-93 and R.214-99 from the French Ministry of Agriculture. Animal House and the responsible party (first author, JFM) received the official agreements # A-31-555-03 and # 31-08-555-17), respectively. Rodent feces were collected thrice a week and then mixed with oxygen-saturated tap water. The resulting paste was dispensed into 10 cm-diameter Petri dishes and incubated at 26 °C. The infective L3 larvae were extracted from the paste using Baermann’s method. A suspension containing approximately 5000 living larvae/mL was prepared. Infections of new batches of rats were initiated by the subcutaneous injection of 250 µL of the larval suspension (1250 L3 larvae per rat).

#### 2.3.3. Immunodiagnosis: IFAT

Whole intact *Strongyloides ratti* filariform larvae were employed since it has been previously demonstrated that the majority of patients with chronic strongyloidiasis had specific lgG antibodies directed against the surface of filariform larvae [28].

Sera were diluted 2-fold in PBS buffer, starting at 1:20 up to 1:160. Fluorescein isothiocyanate-labeled rabbit antihuman IgG (Bio-rad, Marnes-la-Coquette, France) was used as conjugate. The assay was read in a fluorescence microscope (Leitz, Wetzlar, Germany) using X10 oculars and an X20 lens. Internal assessment of the IFAT found that the Se was 77% and the Sp 85% when a dilution of 1:20 was considered the cutoff value (unpublished data). When 1:40 was retained, the Se decreased to 51%, but the Sp increased to 95.8% [19].

IFAT was used in the department from 1990 to 2001 when it was replaced with ELISA.

#### 2.3.4. Immunodiagnosis: ELISA

Clean *S. ratti* infective L3 larvae were transferred to a mortar. They were frozen in liquid nitrogen and crushed by hand with a pestle. The resulting paste was diluted at 1:20 in PBS buffer and submitted to ultrasonic homogenization for 10′ at +4 °C. Extraction of antigenic fractions was performed by overnight magnetic stirring at +4 °C. This crude extract was ultracentrifuged at 25,000× *g* for 30′. The supernatant was collected, dialyzed again with distilled water, and then freeze-dried in 1 mL vials. For every batch of purified extract, the protein concentration was determined with the Pierce BCA Protein Assay Kit **^™^** (Fisher Scientific, Illkirch, France).

For ELISA, a vial containing the lyophilized antigenic extract was added to distilled water. This solution was diluted in Na_2_CO_3_/NaHCO_3_ pH 9.6 buffer to obtain a protein concentration of 4 µg/mL and was distributed in NUNC^®^96-well microtitration plates (Fisher Scientific, Illkirch, France) at a rate of 50 µL per well (80 ng of proteins per well). The following ELISA procedure was conventional. Sera to be tested were diluted at 1:1000 in PBS buffer, and 50 µL were distributed per well. Detection of fixed antibodies was performed with antihuman IgG labeled with horseradish peroxidase (Bio-Rad, Marnes-la-Coquette, France) and TMB substrate (Fisher Scientific, Illkirch, France). The optical density (OD) was read at 450 nm on a Bio Tek™ reader (Agilent, Santa Clara, CA, USA). Following internal assessment of the technique, the Se was 84% and the Sp 92% when the cutoff value was OD 0.410 (unpublished data). Intense cross-reactions were observed in sera from patients presenting with various filariases, mostly due to *Loa-loa* or *Mansonella* spp., pin-worm infection, and cystic echinococcosis. These findings suggested that *Strongyloides stercoralis/S. ratti* shares antigenic fractions with certain Filaria and *Echinoccus granulosus* [29].

### 2.4. Drugs and Treatment Policies

By the end of the 1980s, albendazole (ABZ) appeared to be a good alternative to thiabendazole due to its86% efficacy and lesser degree of side effects [30]. However, obtaining this cure rate required the administration of ABZ at 16 mg/kg b.w. daily for 3 days and the repetition of the treatment 12 days later. At our clinic, this regimen was found to induce rather frequent side effects, particularly nausea or vomiting, so the patients diagnosed with strongyloidiasis were given ABZ 10 mg/kg b.w. daily for 4 days, and the treatment had to be repeated 7 days later. In 1992, ivermectin (IVM) was found to have great efficacy in treating strongyloidiasis patients [31]. The drug became available in France under compassionate gifts and, in 1999, was registered for the treatment of *S. stercoralis* infection. As of 1993, IVM was, therefore, offered to the strongyloidiasis patients who were diagnosed in the clinic. Primarily, the regimen was 200 µg/kg b.w.p.o. in two doses separated by a 1-week interval. Later, administration for two consecutive days was utilized [32]. As long as ABZ or IVM were not registered in France for treatment of strongyloidiasis, oral informed consent was obtained from the patients before therapy.

Regardless of the anthelmintic that was used, the treated patients were required to attend the clinic for a follow-up consultation two months after their treatment had ended.

### 2.5. Statistical Analysis

Statistical analysis included descriptive statistics and the analysis of the variation in 4 parameters following anthelmintic treatment. These variables were the clinical score (see above), the level of blood eosinophilia and serum total IgE, and the result from immunodiagnosis, either by IFAT or ELISA. The statistical package Intercooled Stata™ (StataCorp LLC, College Station, TX, USA) was used for statistical analysis. A comparison of the studied parameters was made by the Wilcoxon signed-rank test or Cochran’s Q test as appropriate. Since the Wilcoxon test is not parametric, only the median and the interquartile range [IQR] values, and not the mean and standard deviation values, of continuous variables were displayed in tables.

## 3. Results

Demographic epidemiological characteristics of the patients are displayed in Table 1. Patients who were born in France and did not report any stay or travel outside the country were considered autochthonous cases.

The likely origin of the infection was classified according to geography regardless of nationality. French citizens from Overseas Territories were therefore included either in the Caribbean region (one case from La Martinique and two from La Guadeloupe islands) or in the Indo-Pacific area (one from Nouvelle-Calédonie and five from La Réunion island). For the three French citizens who became infected during a stay or travel outside France, the visited countries were Zaïre (now the Democratic Republic of the Congo) and Gabon (one case), Kenya and the West Indies (one case), or Vietnam (one case). Concerning the three patients who had been possibly infected in Portugal, the precise area of contamination was not recorded.

Table 2 displays the clinical and laboratory features of strongyloidiasis collected from the 69 attendees. Since strongyloidiasis may persist for decades [1], the period of the onset of clinical manifestations and, therefore, the likely duration of the disease before attendance at the clinic proved to be quite difficult to assess. Consequently, patients’ replies were split into two classes: Three months or less and over three months. Eosinophilia detected by personal physicians was the main reason (81.2%) for referring the patients to the clinic, as investigation of blood eosinophilia was recognized expertise of the Department of Parasitology-Mycology [11].

Table 3 displays the combined results of anthelmintic therapy on the clinical score and laboratory parameters, regardless of the anthelmintic used.

Table 4 displays the results from the statistical analysis of the respective efficacy of ABZ and IVM on the clinical score, eosinophilia level, and serum total IgE level. Although both drugs showed significant action, the values of *p* indicated a greater strength of evidence for IVM efficacy. Concerning the differential kinetics of IFAT or ELISA according to the anthelmintic used, whether ABZ or IVM, too small numbers of results per class were available, and, therefore, a statistical analysis had no interest.

## 4. Discussion

The Outpatient Clinic of the Department of Parasitology-Mycology in Toulouse University Hospitals specializes in the diagnosis and treatment of parasitic diseases, particularly helminthiases, but is not a Tropical Diseases Unit or a Traveler’s Health Clinic. This status explains differences when the present study was compared to surveys that were carried out in Great Britain, Spain, or Switzerland on imported strongyloidiasis cases [33,34,35,36]. Here, we found a low percentage of travelers (4.35%), the presence of autochthonous cases (15.9%), and imported strongyloidiasis in patients from nearby European countries (10.1%) or veterans (8.7%).

Strongyloidiasis is a worldwide helminthiasis that is highly prevalent in tropical or subtropical countries [37]. Additionally, endemic pockets have been recorded in more temperate areas, such as the Southeastern states of the USA [38] or Southern Europe [39]. Concerning France, the presence of autochthonous strongyloidiasis in the Midi-Pyrénées Region had been previously reported [19]. A clear risk factor, namely, frequent contact with soil, was identified in six autochthonous infections out of 11 and was occupational (farming, four cases; pool-installing, one case) or recreational (flower gardening without protective gloves, one case). These findings suggest the persistence in the Midi-Pyrénées Region and particularly in the Garonne River basin of low-intensity transmission of strongyloidiasis. Whether a zoonotic origin of the infection could explain this situation should, therefore, be investigated.

Seven patients were former immigrants from Italy (4) or Portugal (3) who had never traveled outside the European Union. All patients of Italian origin were from Venetia, an area of Northern Italy that was identified in the 1980s as a focus of strongyloidiasis [40]. A multicenter case-control study has recently suggested that this historical focus has been extinguished since *S. stercoralis* infection involved only elderly people living in the studied areas [41]. In support of this statement, the age of our four patients of Italian origin ranged from 64 years old to 85 years old by the time of consultation, between 1991 and 2000.

For the three former immigrants of Portuguese origin, the place of residence in Portugal was not identified. However, a retrospective study reported that the central region in Portugal surrounding the town of Coimbra was the historical focus of autochthonous strongyloidiasis in this country [42].

A wet tropical climate, along with the agricultural habit of rice paddies, placed Southeast Asia and particularly Vietnam among the most prevalent areas for strongyloidiasis worldwide [43]. Not surprisingly, *S. stercoralis* infection was recorded in cohorts of Australian or US veterans from the Vietnam War [44,45]. However, the operations during the so-called Indochina War (aka the 1st Vietnam War) that took place from 1947 to 1954 had to wait until 2017 to be officially recognized as a risk factor for strongyloidiasis in French veterans. The four-decade time gap between the end of the war and the attendance at the clinic underlines the known persistence of strongyloidiasis in infected subjects [1].

Sixteen patients out of 69 (23.2%) were asymptomatic. This feature of chronic strongyloidiasis is classic [3] but is usually present at a higher rate (49.6%) according to a recent meta-analysis [46]. A likely cause of this discrepancy could be the recruitment method of our patients. Our clinic acts as a 2nd-line investigative center, the first line being general practitioners (GP) or, rarely, specialists. Obviously, an asymptomatic subject has few reasons to consult a physician. Fourteen patients (20.3%) in our study declared that the signs or symptoms that prompted them to consult their personal doctor arose within three months before the attendance at the clinic. Upon interrogatory and physical examination, their clinical picture was consistent with uncomplicated chronic strongyloidiasis and not with a recent infection. This finding underlines a classic characteristic of *S. stercoralis* infection, namely, the occurrence of clinical manifestations a long period after contamination [3].

The collected clinical data (Table 2) formed the classic triad of abdominal pain, diarrhea, and skin allergy. The prominence of the latter signs or symptoms was consistent with the findings from Buonfrate et al.’s meta-analysis [46], but the frequencies were lower in the present study: 11.6% vs. 33.8% for itching (pruritus *sine material*) and 15.9% vs. 29.7% for other signs of skin allergy, particularly urticaria. Moreover, nausea/vomiting or respiratory signs or symptoms were not recorded in the present study, whereas these manifestations were frequently reported in cases from endemic areas. The presence in our study of one patient out of four who was infected in nonendemic countries of Southern Europe could explain this discrepancy.

Blood eosinophilia and the level of serum total IgE were the studied nonspecific laboratory parameters. Table 2 indicates that the frequency of blood eosinophilia was affected by a recruitment bias since, for 81.2% (56) of the patients, this hematological abnormality was the main reason to be referred to the clinic. However, only 76.8% (53) had an eosinophil count result ≥ 0.5 G/L on their first visit, which underlines the intermittent nature of eosinophilia in strongyloidiasis [10]. According to the current categories, the median eosinophilia level (Table 2) may be coined “mild” and is in accordance with the findings from the above-cited meta-analysis (median values from 0.9 G/L to 1.35 G/L) [46]. A rise in total IgE levels in patients chronically infected by worms has been primarily reported in Ethiopian children with ascariasis [47] and in most helminthiases, including strongyloidiasis [1,9,36]. The pathogenesis of this increase remains partially unknown. In cases of strongyloidiasis, the mean level of serum total IgE has varied greatly between studies, ranging from 311 [48] to 1364 kIU/L [9]. Different techniques for the dosage of total IgE can partially explain these scattered results, but the patient’s epidemiological status could also be responsible. In a Spanish survey [36], the mean level of serum total IgE was 58 kIU/L in travelers, namely, inside the normal range, vs. 642 kIU/L in immigrants from endemic countries, thus suggesting that the age of the infection would be correlated with the level of this class of immunoglobulin. In Table 2, the median level of 650 kIU/L for total IgE combined with a duration of the infection ranging from >3 months to 40 years in 68.1% of the patients supports this hypothesis.

To date, the better efficacy of IVM for treating *S. stercoralis* infection has been clearly established [49]. The present study, therefore, was not a therapeutic trial and was intended only to compare the posttreatment kinetics of the clinical score and laboratory parameters. Kinetics was first analyzed (Table 2) regardless of the anthelmintic drug used (global analysis). Then, the patients were sorted according to the therapy, ABZ vs. IVM, and the results, except those from immunodiagnosis (see above), were analyzed (Table 4). The results of both assessments were similar, and a general trend was disclosed. Within six months, the level of the clinical score dropped off, and this result is in accordance with the above-cited meta-analysis [46]. This study found that the frequency of all symptoms was reduced compared to baseline. The median eosinophil count normalized under 0.5 G/L in both the global assessment of the kinetics and in the IVM arm. The kinetics of serum total IgE appeared to be constantly slow. Experimental studies have been carried out either in healthy subjects or in patients with allergic diseases, consisting of the infusion of a solution of human IgE. In healthy subjects, the serum concentration of total IgE was reduced by half within three days, but the decay curve became flat, and the levels at day 6 and day 50 were similar [50]. In three strongyloidiasis patients treated and cured by ivermectin in Northern France, the total IgE level remained far over the upper value of the normal range at the end of a three-year follow-up [51]. Consequently, the serum total IgE appeared to be useless for the monitoring of treated strongyloidiasis patients, whereas this measurement plays a pivotal role during the investigation of blood eosinophilia [11].

Either at the diagnostic step or for the posttreatment follow-up, ELISA based upon a purified antigenic extract of *S. ratti* filariform larvae showed a clear advantage in terms of Se over IFAT using whole filariform larvae of the same species. This fact is a further and retrospective justification of our discontinuation of IFAT and the switch to ELISA by the turn of the 2000s.

## 5. Conclusions

The present study, which involved the scrutinization of 69 file records of patients infected with *S. stercoralis*, confirms the presence of the autochthonous transmission of strongyloidiasis in Southwestern France. Additionally, this study stresses the reasons why strongyloidiasis is still a neglected disease in developed countries such as France. The autoinfection route resulting in the permanent presence of the parasite in the human body is a fact that remains ignored by most GP or specialists. Moreover, strongyloidiasis shares with some other helminthiases a nonspecific clinical picture.

Due to these diagnostic issues, we have chosen through our postgraduate sessions to suggest to GP or specialized physicians that they systematically investigate any case of blood eosinophilia, regardless of the origin and/or the medical history of the patient. However, a further difficulty concerning the laboratory diagnosis of strongyloidiasis by stool examination occurred in France during the decade 2005–2015. By that time, the legal obligation of accreditation for the structures performing medical analyses induced changes in the organization of the private laboratories. Concerning stool examination, a consequence was Baermann’s method, or agar-plate culture became very difficult to perform routinely. Meanwhile, an increasing number of prescribers neglected stool examination and ordered only specific serology. However, molecular diagnosis of *S. stercoralis* infection, which was introduced and has since then been improved continuously, will certainly represent a solution to this diagnostic issue.

In conclusion, we believe that the resolution of clinical features, such as assessed by the use of a scoring system, and the kinetics of eosinophilia are the most appropriate parameters to check during the posttreatment follow-up.

## Figures and Tables

**Table 1:** Demographic and epidemiological characteristics of 69 strongyloidiasis cases diagnosed at the outpatient clinic.

*Age (Years)*	
Median [IQR ^1^]	40 [25–57]
Classes, % (*n*)	
1–7	11.6 (8)
8–18	7.3 (5)
19–49	47.8 (33)
≥50	33.3 (23)
*Sex*, % (*n*)	
Females	37.7 (26)
Males	62.3 (43)
*Residence, % (n)*	
Rural or towns ≤ 5000 inhabitants	30.4 (21)
Towns > 5000 inhabitants	26.1 (18)
Toulouse conurbation	43.5 (30)
*Autochthonous cases, % (n)*	15.9 (11)
*Former service members, Indochina war*	
*or Pacific war, % (n)*	8.7 (6)
*Immigrants from endemic areas outside European Union, % (n)*	
East Africa ^2^	4.35 (3)
North Africa ^3^	2.9 (2)
Sub-Saharan Africa ^4^	18.85 (13)
Caribbean and South America ^5^	14.5 (10)
Indopacific area ^6^	20.3 (14)
*Immigrants from the European Union, % (n)*	
Portugal	4.35 (3)
Italia (Venetia)	5.8 (4)
*Infection likely acquired during a stay or travel outside France, % (n)*	4.35 (3)
*Occupational exposure to strongyloidiasis*, % (*n*) ^7^	17.4 (12)
Construction and Related Workers, Pool Installer	1.45 (1)
Construction Laborer	1.45 (1)
Farmers, Ranchers, and Other Agricultural Managers	7.25 (5)
First-Line Supervisor of Construction and Extraction Workers	1.45 (1)
Stonemasons	5.8 (4)

^1^: Interquartile range; ^2^: Ethiopia (3); ^3^: Algeria (1), Morocco (1); ^4^: Angola (3), Congo, (1), Ghana (1), Ivory Coast (1), Mali (1), Republic of Central Africa (2), Senegal (1), Togo (1), Zaïre (2); ^5^: Brazil (1), Guadeloupe (2), Haiti (5), La Martinique (1); ^6^: Cambodia (2), Comoros Islands (2), Nouvelle-Calédonie (1), La Réunion (5), Laos (3), Vietnam (1); ^7^: Standard Occupational Classification, U.S. Bureau of Labor Statistics.

**Table 2:** Clinical and laboratory features of strongyloidiasis in 69 cases diagnosed at the outpatient clinic.

*Duration of the Clinical Picture before Attendance at the Clinic, % (n)*	
Not available	11.6 (8)
≤3 months	20.3 (14)
From >3 months to 40 years	68.1 (47)
*Primary reason for attending the clinic, % (n)*	
Eosinophilia associated with various clinical signs or symptoms	37.7 (26)
Eosinophilia found during a health check-up	14.5 (10)
Eosinophilia found during investigation of a noninfectious disorder	5.8 (4)
Eosinophilia in a patient originating from outside the European Union	8.7 (6)
Health check-up in adopted children or refugees	14.5 (10)
Health check-up following stay or travel in/to a tropical area	4.3 (3)
Longstanding eosinophilia	14.5 (10)
*Clinical signs or symptoms recorded at the first consultation, % (n)*	
Asymptomatic	23.2 (16)
Arthralgia and/or myalgia	7.25 (5)
Bloating/colic pain	10.1 (7)
Chronic irritating cough	1.45 (1)
Conjunctivitis	2.9 (2)
Gastric pain	15.9 (11)
Intermittent diarrhea	18.8 (13)
*Larva currens*	1.45 (1)
Otorhinolaryngeal allergy manifestations ^1^	4.3 (3)
Pruritus *sine materia*	11.6 (8)
Skin allergy manifestations ^2^	15.9 (11)
Weakness	13.1 (9)
*Clinical score * ^3^ * in 53 symptomatic patients, median [IQR] * ^4^	2 [2–4}
*Laboratory parameters*	
*Continuous, median [IQR]* ^4^	
Eosinophil count (G/L) ^5^	1.0 [0.5–1.75]
Serum total IgE (kIU/L) ^6^	540 [168–1456]
Immunodiagnosis by ELISA in 18 patients (OD ^7^)	1.2 [0.94–1.5]
*Categorical, % (n)*	
Positive results from ELISA in 18 patients ^7^	88.9 (16)
Positive results from indirect immunofluorescence in 51 patients ^8^	
≥20	74.5 (38)
≥40	52.9 (27)

^1^: Rhinorrhea, nasal congestion, sneezing; ^2^: Eczema, itchy rashes, urticaria; ^3^: Sign or symptom: Present, 2; absent, 0; ^4^: Interquartile range; ^5^: Giga cells per liter; ^6^: Kilo international units per liter; normal values ≤ 150 kilo International Units/L; ^7^: Optical densities; cutoff value: OD 0.410; ^8^: Reciprocal value of serum dilutions.

**Table 3:** Global kinetics of clinical and laboratory parameters in 48 patients who attended the follow-up consultation.

*Interval * ^1^ * (Weeks), Median [IQR] * ^2^			9.5 [6.9–17.3]
*Clinical score * ^3^ * in 32 symptomatic patients, median [IQR] * ^2^			
Before treatment			2 [2–4]
At follow-up consultation			0 [0–1]
*p* ^4^			<0.00001
*Eosinophil count (G/L) * ^5^ *, median [IQR] * ^2^			
Before treatment			1.1 [0.6–1.9]
At follow-up consultation			0.4 [0.2–0.65]
*p * ^4^			<0.00001
*Serum total IgE (kIU/L) * ^6^ *, median [IQR] * ^2^			
Before treatment			650 [201–1490]
At follow-up consultation			532 [133–1135]
*p* ^4^			0.0002
*Immunodiagnosis by ELISA in 15 patients (OD) *^7^*, median [IQR] * ^2^			
Before treatment			1.2 [0.84–1.47]
At follow-up consultation			0.57 [0.71–1.14]
*p* ^4^			0.0035
*Immunodiagnosis by indirect immunofluorescence in 34 patients*			
Class ^8^	Before treatment, % (*n*)	At follow-up consultation, % (*n*)	
<20	26.5 (9)	41.2 (14)	
20	17.6 (6)	17.6 (6)	*p * ^9^
40	32.4 (11)	29.4 (10)	0.0134
80	14.7 (5)	5.9 (2)	
160	8.8 (3)	5.9 (2)	

^1^: Interval between the end of therapy and the follow-up consultation; ^2^: Interquartile range; ^3^: Pairs of negative results excluded; ^4^: Wilcoxon’s signed-rank test; ^5^: Giga cells per liter; ^6^: Kilo international units per liter: Normal values ≤ 150 kIU/L; ^7^: Optical densities; ^8^: Reciprocal values of dilutions; ^9^: Cochran’s *Q* test.

**Table 4:** Kinetics of clinical and laboratory parameters recorded from patients treated with albendazole (*n* = 11) or with ivermectin (*n* = 37).

	Median [IQR] ^1^		
*Interval (weeks) * ^2^			
**ABZ** ^3^	9.6 [5.9–22.6]		
**IVM** ^4^	9.3 [6.9–16.1]		
	Before treatment	After treatment	*P * ^5^
*Clinical score * ^6^			
**ABZ** ^3^	3 [2–4]	1 [0–1]	0.0251
**IVM** ^4^	2 [2–4]	0 [0–1]	0.00016
*Eosinophil count (cells G/L)*			
**ABZ** ^3^	1.4 [0.6–1.8]	0.5 [0.2–1.0]	0.01
**IVM** ^4^	1.1 [0.6–2.0]	0.3 [0.15–0.6]	<0.00001
*Serum total IgE * ^7^ * (kIU/L)*			
**ABZ** ^3^	1042 [303–3180]	818 [233–2540]	NS ^8^
**IVM** ^4^	642 [206–1456]	515 [115–928]	0.00022

^1^: Interquartile range; ^2^: Interval between the end of therapy and the follow-up consultation; ^3^: Albendazole; ^4^: Ivermectin; ^5^: Wilcoxon’s signed-rank test; ^6^: Asymptomatic patients (pairs of negative results) excluded; ^7^: Normal values ≤ 150 kilo International Units/L; ^8^: Not significant.

## Data Availability

The data presented in this study are available on request from the corresponding author. The data are not publicly available due to legal restrictions.
